# Supplementary material for: Mutational dissection of a hole hopping route in a lytic polysaccharide monooxygenase (LPMO)
Source: Nat Commun. 2024 May 10;15:3975. doi: 10.1038/s41467-024-48245-w (PMC11087555; doi:10.1038/s41467-024-48245-w)
Supplement: Supplementary file 1 — Supplementary Information [file 41467_2024_48245_MOESM1_ESM.pdf]

Supplementary information for

Mutational dissection of a hole hopping route in a lytic polysaccharide monooxygenase (LPMO)

Ivan Ayuso-Fernandez[(1)*], Tom Z Emrich-Mills[(1)], Julia Haak[(2,3)], Ole Golten[(1)], Kelsi R. Hall[(1)], Lorenz Schwaiger[(4)], Trond S. Moe[(1)], Anton A. Stepnov[(1)], Roland Ludwig[(4)], George E. Cutsail III[(2,3)], Morten Sørlie[(1)], Åsmund Kjendseth Røhr[(1)], Vincent G.H. Eijsink[(1)*]

[1] Faculty of Chemistry, Biotechnology and Food Science, Norwegian University of Life Sciences (NMBU), 1432 Ås, Norway.

[2] Max Planck Institute for Chemical Energy Conversion, Stiftstrasse 34-36, 45470 Mülheim an der Ruhr, Germany.

[3] Institute of Inorganic Chemistry, University of Duisburg-Essen, 45141 Essen, Germany

[4] Biocatalysis and Biosensing Laboratory, Department of Food Sciences and Technology, Institute of Food Science and Technology, University of Natural Resources and Life Sciences (BOKU), Muthgasse 18/2, Vienna 1190, Austria

*e-mail: ivan.ayuso-fernandez@nmbu.no or vincent.eijsink@nmbu.no

Contents

Supplementary methods 3

Melting temperature 3

Substrate binding 3

H_2O_2 Production Assay 4

Product analysis by MALDI-TOF MS 4

Real-time monitoring of H_2O_2 turnover 4

Supplementary results & discussion 7

Supplementary Figure 1. Phylogenetic analysis and trees for AA10 LPMOs 8

Supplementary Figure 2. pH, reductant and temperature dependence of radical formation for SmAA10A-Cu(I) 9

Supplementary Figure 3. H_2O_2 dependence of radical formation for *Sm*AA10A 10

Supplementary Figure 4. Time courses for oxidation of β-chitin 11

Supplementary Figure 5. Spectral subtractions for WT SmAA10A and its W178 variants. 12

Supplementary Figure 6. UV-vis absorption traces for *Sm*AA10A variants................ 13

Supplementary Figure 7. Radical formation in the H160F variant. 14

Supplementary Figure 8. Melting temperatures of the different variants of *Sm*AA10A. 15

Supplementary Figure 9. Time courses for binding of WT *Sm*AA10A and its variants to ß-chitin 16

Supplementary Figure 10. Oxidase activity of *Sm*AA10A variants (2 μM).................. 17

Supplementary Figure 11. MALDI-TOF analysis of chitin oxidation by wild-type *Sm*AA10A and the W119F variant.. 18

Supplementary Figure 12. Kinetic characterization of the W119F variant of *Sm*AA10A using an H_2O_2 sensor and stopped-flow fluorescence spectroscopy 19

Supplementary Figure 13: EPR spectrum of wild type enzyme 21

Supplementary Figure 14: EPR spectra of SmAA10A and its variants. 23

Supplementary Figure 15. The environment of H160 and W119 in SmAA10A WT. ... 24

Supplementary Table 1. Strategy to generate each variant gene and list of primers used in this study 25

Supplementary references 26

Supplementary methods

Melting temperature

SYPRO® orange dye from Invitrogen was used to monitor the apparent melting temperature (T_m) of LPMOs[1]. Fluorescence by this dye is naturally quenched and is enhanced when it binds to hydrophobic residues of proteins that become exposed upon unfolding. A temperature ramp from 25 to 98 ºC with an increase of 1.5 ºC/min was used to evaluate the T_m. The SYPRO® orange stock solution (5000x) was diluted to an 8x stock in Milli-Q H_2O prior to adding it for 1x working concentration in the reaction. The reactions contained 30 µM Cu^{2+}-saturated LPMO and 1x SYPRO® Orange dye in 50 mM Tris pH 7.0. Reactions were prepared in quadruplicates, including control reactions without enzyme. The fluorescence change was monitored with a StepOnePlus™ Real-Time PCR (ThermoFisher Scientific). The StepOnePlus™ software (v2.3) was used to obtain the negative first derivative of the normalized fluorescence signal with respect to temperature ($-dF/dT$), allowing an easy identification of the Tm as the lowest -dF/dT peak.

Substrate binding

Binding to ß-chitin was evaluated under conditions similar to those used for LPMO activity measurements. Reactions were carried out at 22 ºC, with 1 mM ascorbate present to analyze the binding capacity of LPMO-Cu(I). 5 µM enzyme was added to mixtures of 10 g/L of ß-chitin in 50 mM Tris, pH 8.0, with 1 mM ascorbate supplemented immediately before the addition of the LPMO. Samples were taken after 1, 3, 5, 10, 15, 30 and 60 minutes of incubation, and filtered using a 96-well filter plate (Merck) operated with a vacuum manifold (Millipore). The unbound protein present in the soluble fraction was quantified with an adapted Bradford protocol for measuring low protein concentrations[2]. For each *Sm*AA10A variant, a control reaction without ß-chitin was included and used as reference to calculate the fraction (%) of unbound protein. Controls without

enzyme were also added to monitor unspecific signals in the Bradford assay. All reactions were done in triplicates.

H_2O_2 Production Assay

H_2O_2 production was measured as previously described[3]. Stock solutions (10 mM) of Amplex™ Red Reagent (Thermo Fisher Scientific, Waltham, MA, USA) were prepared in DMSO. Reactions were prepared in a 90 µL volume containing 50 mM Tris, pH 8.0, 100 µM Amplex™ Red Reagent, 5 U/mL horseradish peroxidase (HRP, Sigma) and 2 µM LPMO, and pre-incubated at 30 °C for 5 minutes. Following pre-incubation, reactions were initiated with the addition of 10 µL of 10 mM ascorbate (1 mM final concentration) and the reaction mixtures were incubated at 30 °C. Formation of resorufin was monitored over 40 minutes at 540 nm in a Multiskan™ FC microplate photometer (Thermo Fisher Scientific, Waltham, MA, USA). A H_2O_2 standard curve was prepared in the same manner, with ascorbate added prior to the addition of Amplex™ Red Reagent and HRP[4].

Product analysis by MALDI-TOF MS

Soluble products of β-chitin degradation by *Sm*AA10A or its W119F variant were analyzed using a matrix-assisted laser desorption/ionization time-of-flight (MALDI-TOF) UltrafleXtreme mass spectrometer (Bruker Daltonics GmbH, Bremen, Germany). 1 µl of the filtered reaction sample was mixed with 2 µl of a matrix solution [9 mg/ml 2,5-dihydrooxybenzoic acid in 30% (v/v) acetonitrile] on the surface of an MTP 384-ground steel target plate (Bruker Daltonics). The target plate was air-dried, and MS data were collected using Bruker flexControl (version 3.4, build 169.5) software as described previously[5].

Real-time monitoring of H_2O_2 turnover

The construction, use and experimental potential of the H_2O_2 sensor are described in detail in Schwaiger *et al.*[6]. The H_2O_2 sensor is based on the fast electrochemical detection of H_2O_2 using an Autolab potentiostat (PGSTAT101, Metrohm) connected to a rotating disk module (motor controller and rotating disk-setup, AUT.RDE.S, Metrohm). The measurements were performed using a three-electrode setup consisting of a gold rotating disk electrode (RDE,

RDE.AU50.S, d = 5 mm, Metrohm) as the working electrode (WE), a coiled platinum wire (BASi) as the counter electrode (CE), and a Ag|AgCl electrode (3 M KCl, MF-2056, BASi) as the reference electrode (RE). The RE was protected from substrate poisoning by using a glass double junction (MF-2030, BASi) filled with 100 mM KCl. The stability of the reference electrode was checked on a regular basis by measuring the potential against the Labmaster electrode (EF-1352, BASi). The potential difference between the reference electrode and the Labmaster electrode is determined by measuring the open circuit potential, i.e. the equilibrium potential developed between the RE and the Labmaster electrode. To increase the sensitivity of the gold rotating disk working electrode, a thin layer of Prussian blue was deposited using cyclic voltammetry. The Prussian blue film is deposited on the surface of the electrode by cycling the WE eight to twelve times in a solution of 2.5 mM $FeCl_3$, 2.5 mM $K_3[Fe(CN)_6]$, 0.1 M KCl, and 0.1 M HCl in a potential window between 600–900 mV vs. SHE at a scan rate of 20 mV s^{-1}. After this deposition step, the WE was thoroughly rinsed with MilliQ-H_2O and activated by electrochemical cycling in a solution of 0.1 M HCl and 0.1 M KCl between 160–590 mV vs. SHE at a scan rate of 50 mV s^{-1}. The activated sensors were then rinsed with MilliQ-H_2O, dried under a stream of N_2, coated with 7 µL of Nafion (Merck, Darmstadt) and cured overnight at ambient atmosphere. On the next day, the prepared sensor was conditioned in the working buffer (50 mM Tris, pH 8.0 & 100 mM KCl) using the same conditions as used in the activation step. All measurements were performed using a water-jacketed low volume cell (MR-1212, BASi) connected to an SE-12 heating circulator (Julabo) to maintain a constant temperature of 37 °C. The H_2O_2 sensor was operated at an applied potential of 100 mV vs. SHE.

All experiments with the H_2O_2 sensor were performed with a volume of 4 mL and typically consisted of 3 essential steps: (i) The baseline was recorded until the signal (current, nA) of the H_2O_2 sensor was constant, then H_2O_2 was titrated into the electrochemical cell in 30 µM steps until a final concentration of 150 µM was reached. For each titration step, the signal was recorded for 30 s. (ii) LPMO was added to a final concentration of 1 µM and the reaction was recorded for another 30s. (iii) The reaction was started by the addition of 50 µM or 1 mM ascorbate

and the time course of the reaction was either recorded until the baseline was reached or until the H_2O_2 time traces flattened out completely before reaching the baseline. The raw data obtained in current (nA) versus time (s) was converted to H_2O_2 concentration (μM) versus time (s) using the calibration function recorded at the start of every single experiment (derived from the initial stepwise titration of H_2O_2). Typically, the H_2O_2 sensors used in this study had sensitivities of 200–250 nA μM^{-1} cm^{-2} and a limit of quantification of 3–4 μM H_2O_2. Initial rates ($\mu M\ s^{-1}$) of reaction were obtained by linear fitting to the initial part of the H_2O_2 depletion curves (usually the first 15 s). Only data based on fits with an R^2 of 0.98 or higher are reported. The data was acquired using the software NOVA 1.1 from Metrohm (Herisau, Switzerland).

Enzyme turnover numbers (TN, s^{-1}) were obtained by subtracting the small but noticeable ascorbate-dependent depletion of H_2O_2 in the presence of 10 g L^{-1} β-chitin from LPMO-catalyzed depletion. This background signal was routinely measured for each measurement series and ranges between 0.06 $\mu M\ s^{-1}$ for 50 μM ascorbate and 0.19 $\mu M\ s^{-1}$ for 1 mM ascorbate. The off-pathway peroxidase-like activity of the LPMO was determined in the absence of substrate, otherwise using the same conditions as defined above. Enzymatic turnover numbers were determined in triplicate, off-pathway peroxidase activity and background H_2O_2 depletion in duplicate.

Supplementary results & discussion

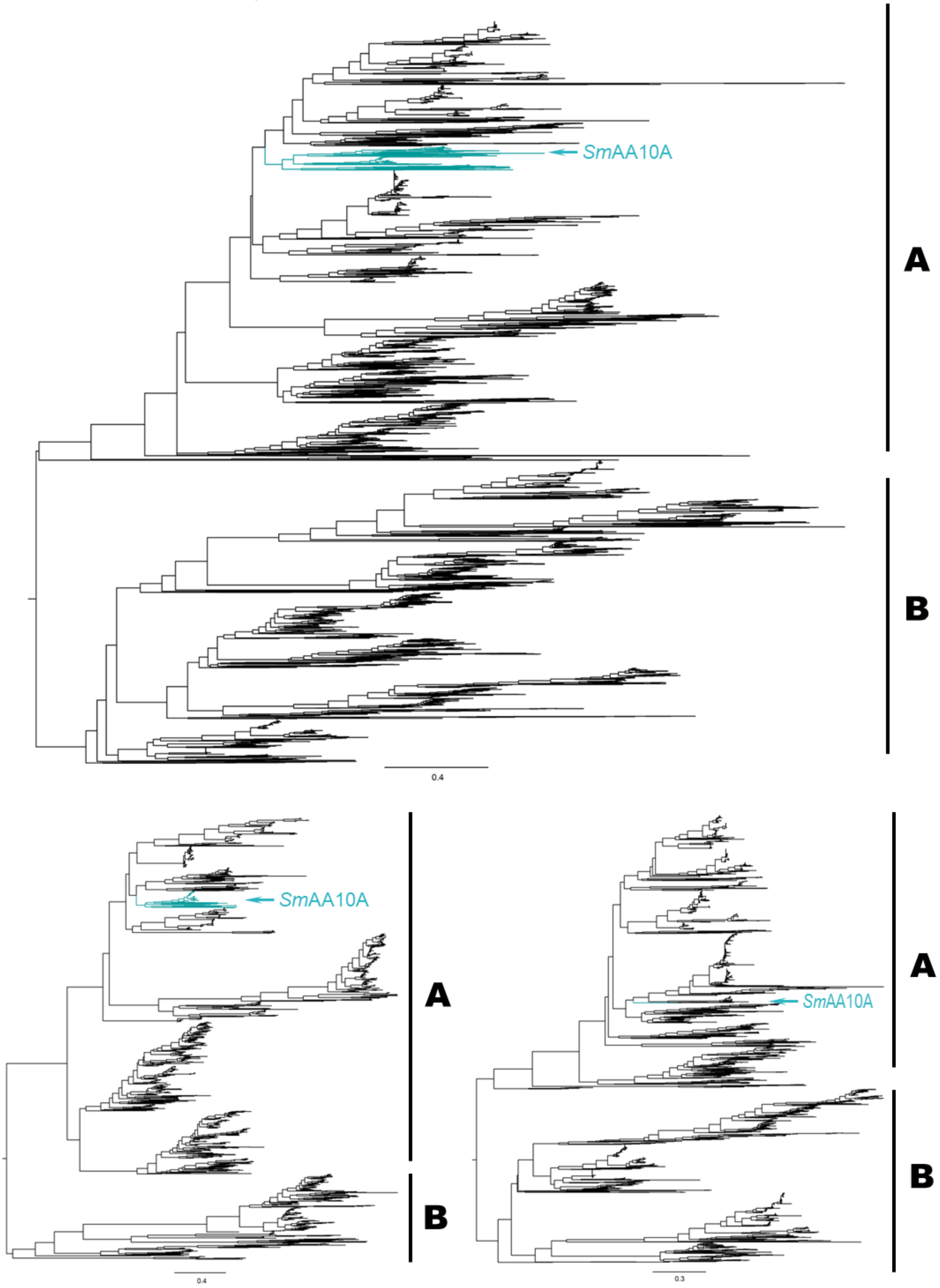

Supplementary Figure 1. Phylogenetic analysis and trees for AA10 LPMOs generated with the three datasets obtained with dbcan_curation.sh. Top: all AA10 LPMOs sequences. Bottom left: AA10 LPMOs annotated as single domain proteins. Bottom right: AA10 LPMOs containing extra domains known as carbohydrate binding modules (CBMs). Only the catalytic domains were used in these analyses. In all cases, we could observe two big clades, **A**, containing chitin-active LPMOs, and **B**, involving containing LPMOs with activity on cellulose only or with mixed (chitin and cellulose) activity. *Sm*AA10A is included as reference, and its subclade is highlighted in blue. Note that in the phylogenetic tree for CBM-containing LPMOs (bottom right), *Sm*AA10A, which is single domain and was artificially added to this tree, appears as a single-sequence branch. After data selection and curation, the sequences were aligned with MAFFT (L-INS-i option) and the trees were obtained using fasttree with default parameters.

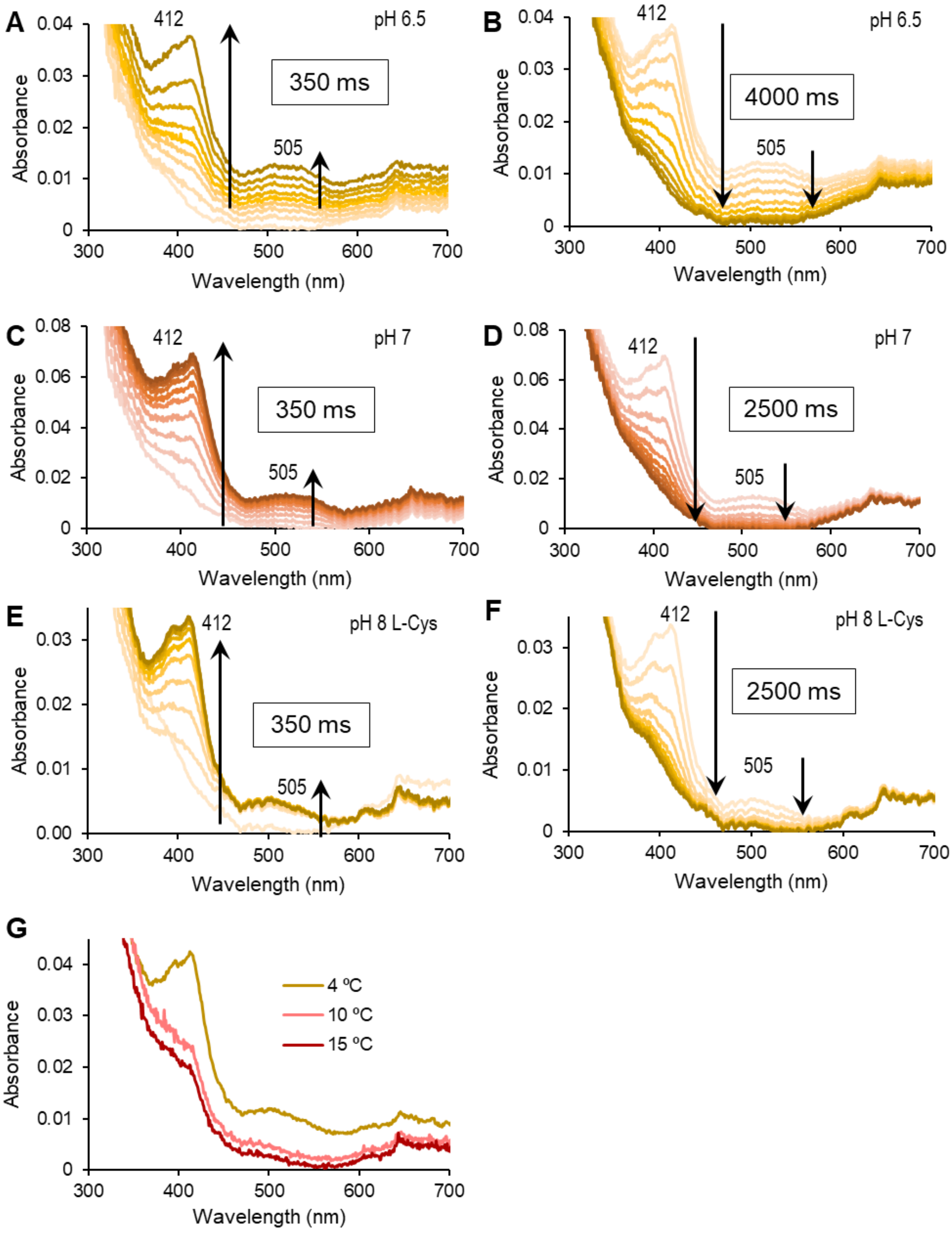

Supplementary Figure 2. pH, reductant and temperature dependence of radical formation for SmAA10A-Cu(I) reacting with 20 molar equivalents of H_2O_2, detected in UV-vis stopped-flow absorption spectroscopy experiments. **A-B:** Bis-Tris, pH 6.5. **C-D:** sodium phosphate, pH 7.0. **E-F:** Tris, pH 8.0. 1 molar equivalent of ascorbate (**A-D**) or L-Cys (**E-F**) was used to generate LPMO-Cu(I) *in situ*. In panels **A** to **F**, the left panels show formation of signals, with the time until the maximum signal was observed indicated in the box. The right panels show the decay of such signals, with the time until full decay indicated in the box. The shapes of the spectra depicted in panels E and F (pH 8.0, L-Cys), as well as the derived rates of feature formation and decay, are very similar to those seen in experiments at pH 8.0 in which ascorbate was used to generate the reduced LPMO (Fig. 2 of the main manuscript). The experiments depicted in panels **A** to **F** were done at 4 °C. Panel **G** illustrates the strong temperature dependence of the signals by showing spectra at maximum signal for the reaction shown for WT in Fig. 2 of the main manuscript, done at three temperatures.

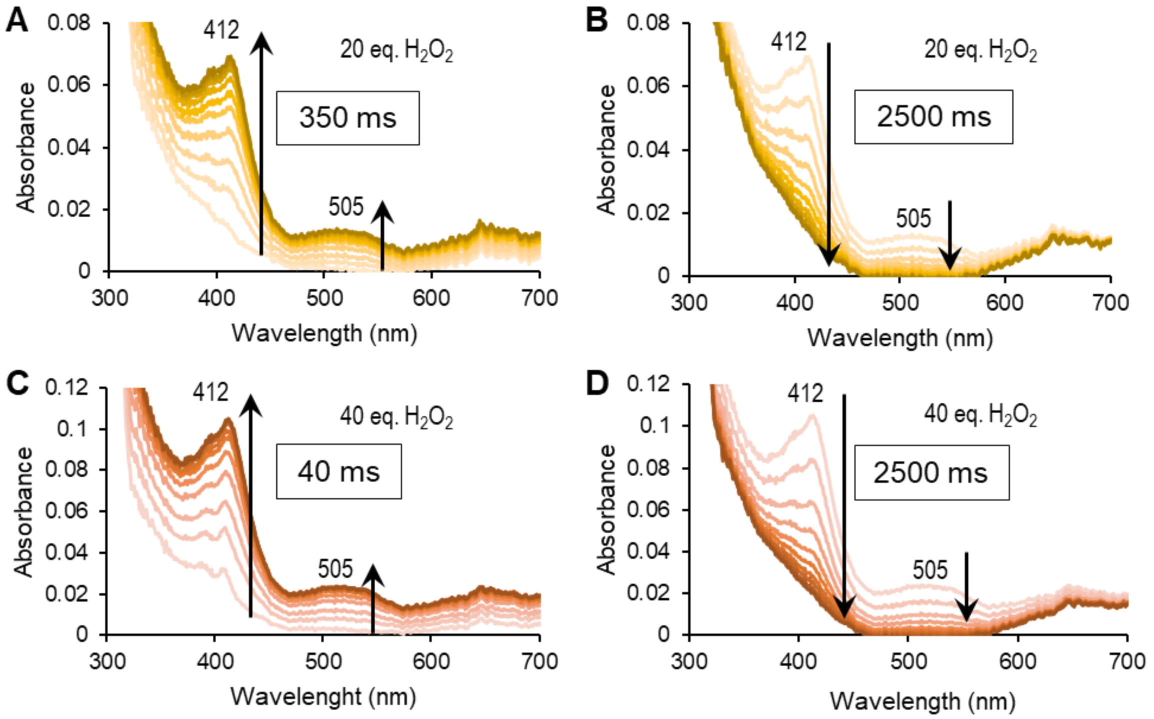

Supplementary Figure 3. H_2O_2 dependence of radical formation for *Sm*AA10A. A-B: experiments with 20 molar equivalents of H_2O_2. **C-D:** experiments with 40 molar equivalents of H_2O_2. Left panels show development of the UV-Vis absorption signals while right panels show decay until resting state, with the times needed to reach maximum signals and full decay indicated in boxes. Note that, while the generation of signals differs between 20 and 40 m. eq. of H_2O_2 (9-fold faster in **C** than in **A**), the decay to resting state happens similarly fast. The reactions were performed at 4 °C in 50 mM sodium phosphate, pH 7.0, using 1 molar equivalent of ascorbate to generate LPMO-Cu(I) *in situ*.

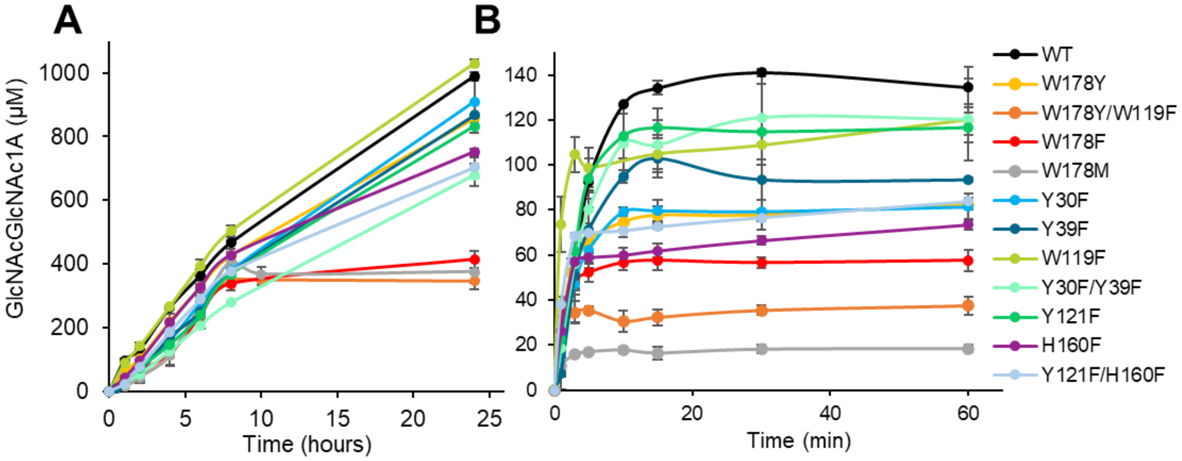

Supplementary Figure 4. Time courses for oxidation of β-chitin. **A:** experiments with no external source of H_2O_2 and 1 mM ascorbate as reductant ("monooxygenase conditions")**. B:** reactions containing 300 µM H_2O_2 and 50 µM ascorbate as reductant ("peroxygenase conditions"). The enzyme concentration was 1 µM in all reactions. Quantification of solubilized oxidized products was done by converting these to chitobionic acid, which was quantified. Reactions were carried out in 50 mM Tris, pH 8.0. Data are represented as mean values ± s.d (n = 3).

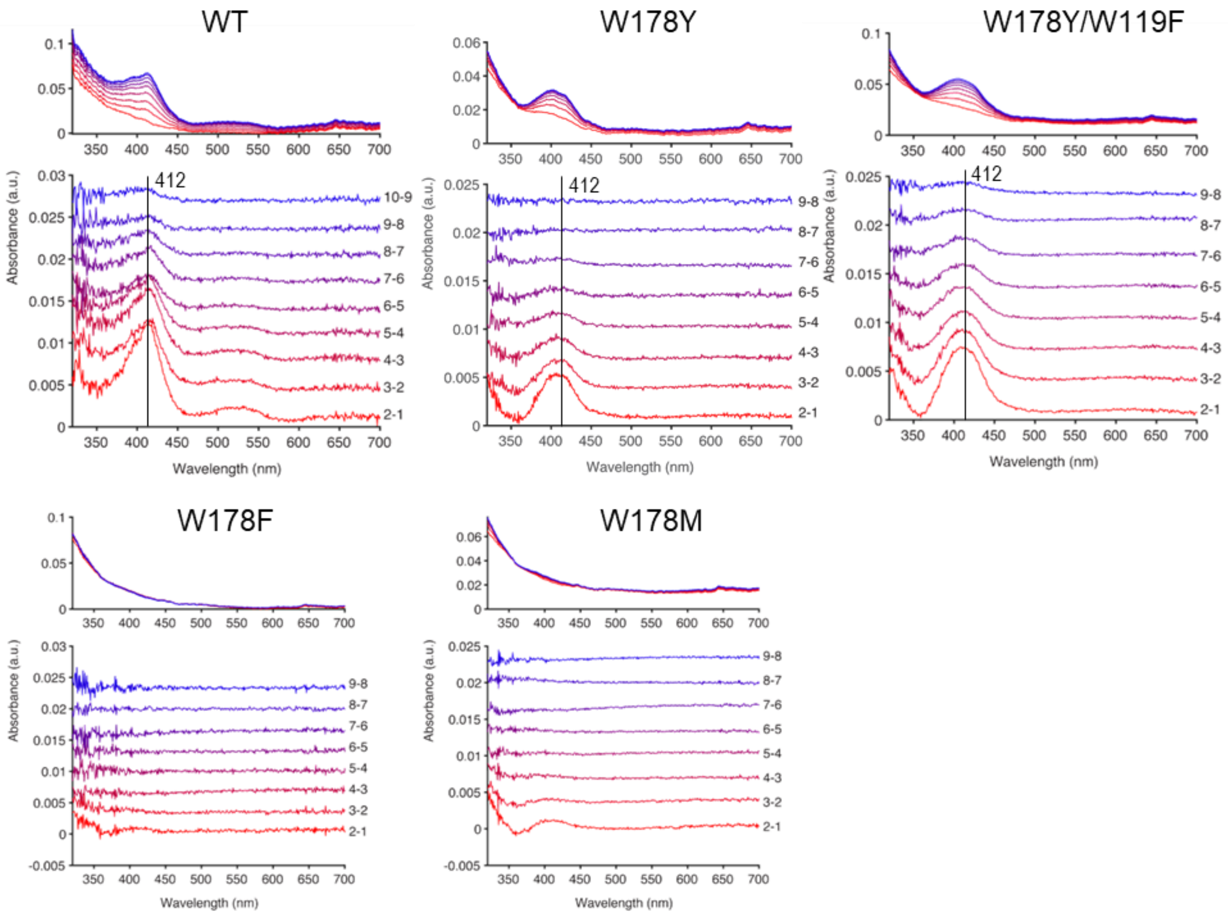

Supplementary Figure 5. Spectral subtractions for WT SmAA10A and its W178 variants. Spectral subtraction was performed on the spectra depicted in Fig. 2 of the main manuscript that show formation of features until maximum signal. There were nine or ten spectra, labeled from 1 (early; the first one) to 9 or 10 (late, the last one). The data were processed by subtracting the preceding spectrum from each of the spectra, as indicated in the Figure. Red color indicates early time points and blue color indicates later time points, with diminishing growth of the signals. The spectra have been vertically offset for better visual presentation of the data. The data for WT *Sm*AA10A show features for both Trp• and Tyr• radicals are. The Trp• feature appears to saturate more quickly, leaving only a Tyr• radical signal in the difference spectra for the later time points. When W178 is mutated to Tyr in the W178Y containing variants, only a sharp, but broader, Tyr•-like feature is observed at a similar maximum wavelength than WT. In variants containing a Phe or a Met instead of Y178, a minute putative Y• feature became visible upon spectral subtraction.

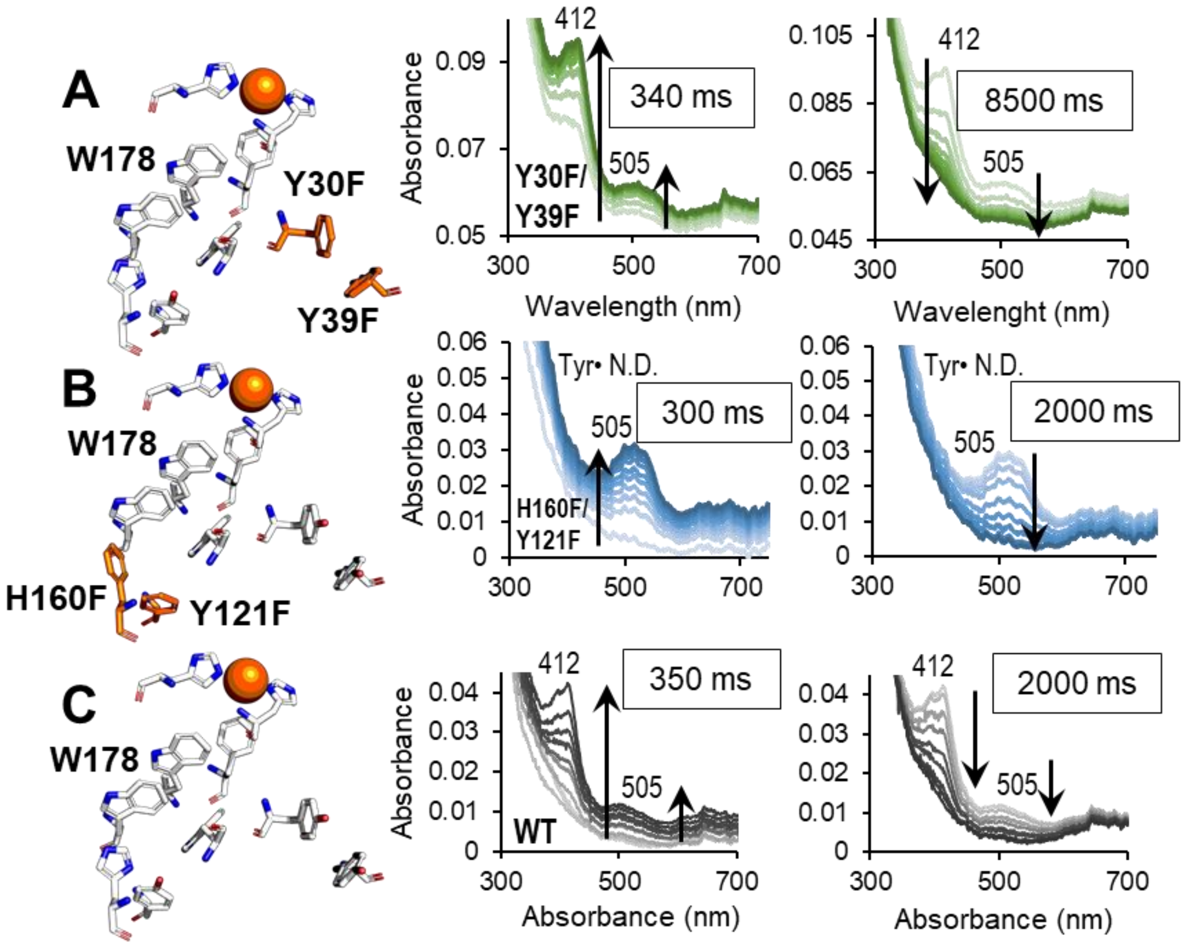

Supplementary Figure 6. UV-vis absorption traces for *Sm*AA10A variants Y30F/Y39F (**A**), Y121F/H160F (**B**) and WT (**C**) reacting with 20 molar equivalents of H_2O_2. Left panels show development of the traces until maximum signal and right panels show decay, with the times needed to reach maximum signals and full decay indicated in boxes. Note that there is no tyrosyl radical feature in panel B, as indicated (N.D not detected). The reactions were performed at 4 °C in 50 mM Tris, pH 8.0, using 1 molar equivalent of ascorbate to generate LPMO-Cu(I) *in situ*.

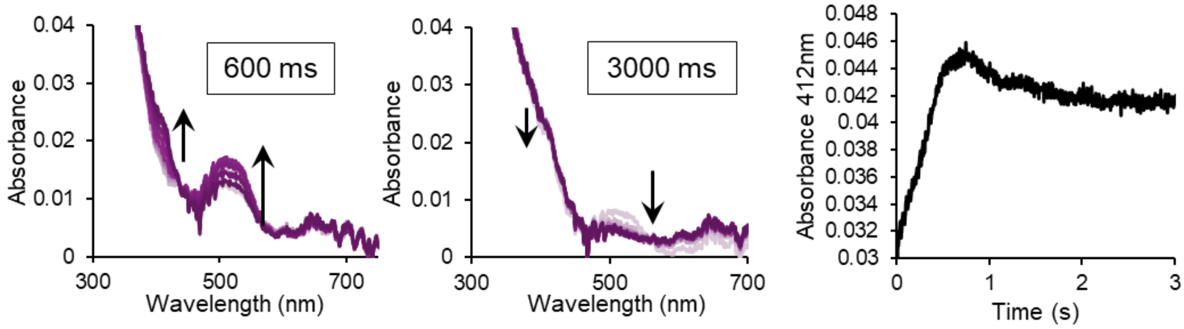

Supplementary Figure 7. Radical formation in the H160F variant. These spectra are from the same experiment as in Fig. 3 of the main manuscript, but the presentation is focused on a longer time scale of formation and decay in an attempt to capture any tyrosyl feature (412 nm). The experimental conditions and the annotation of the left two panels is as in Supplementary Fig. 6. The tryptophanyl feature is clearly observable around 500 nm, but the tyrosyl feature is almost absent despite the presence of Y121, which is likely the major contributor to the tyrosyl signal (see main text and Fig. 3). The left graph shows slow formation of a very weak tyrosyl radical signal, where as the middle graph essentially shows no decay of this signal, as summarized in panel C, showing the 412 nm feature over time. This indicates that H160, on the surface of *Sm*AA10A, is necessary to estabilise and dissipate radicals at Y121.

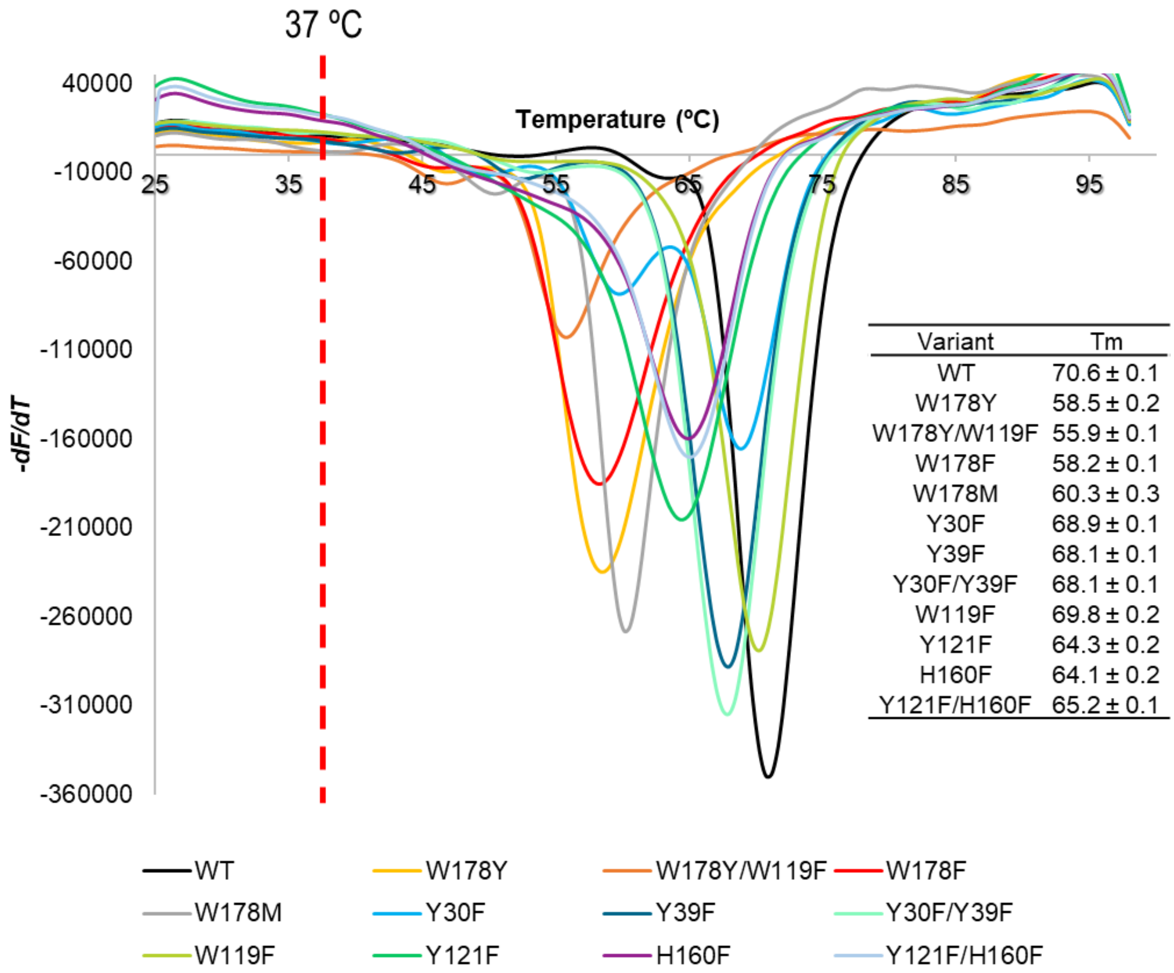

Variant	Tm
WT	70.6 ± 0.1
W178Y	58.5 ± 0.2
W178Y/W119F	55.9 ± 0.1
W178F	58.2 ± 0.1
W178M	60.3 ± 0.3
Y30F	68.9 ± 0.1
Y39F	68.1 ± 0.1
Y30F/Y39F	68.1 ± 0.1
W119F	69.8 ± 0.2
Y121F	64.3 ± 0.2
H160F	64.1 ± 0.2
Y121F/H160F	65.2 ± 0.1

Supplementary Figure 8. Melting temperatures of the different variants of *Sm*AA10A. The SyproOrange® assay was used to determine the apparent Tm, which is the temperature at which dF/dT reaches its maximum. Reactions were carried out in 50 mM Tris, pH 7.0, using 30 µM LPMO, in quadruplicates. The plot represents the mean value. Apparent Tm values, as means ± s.d (n = 4), are shown in the Table.

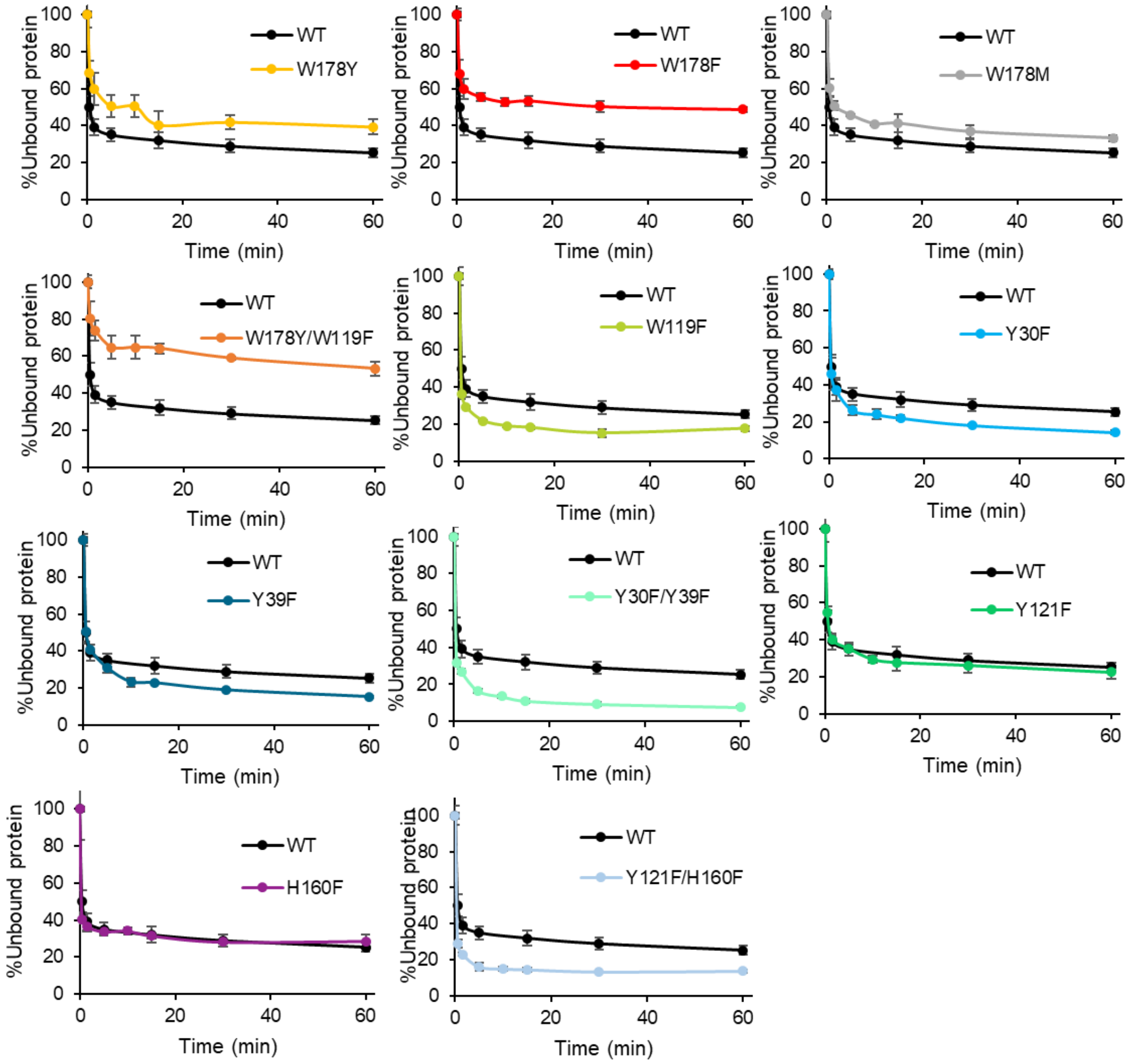

Supplementary Figure 9. Time courses for binding of WT *Sm*AA10A and its variants to β-chitin. To ensure LPMO-Cu(I), 1 mM ascorbate was added to the reaction. Reaction conditions were identical to activity assays (Figs. 2 and 3 of main manuscript, Supplementary Fig. 4A) but were performed at 22 °C to capture binding, while reducing catalysis. The fraction of unbound protein was determined by comparing the measured levels of unbound protein to the level of non-bound protein in control reactions without β-chitin. Reaction samples were collected and filtered after 0.5, 1.5, 5, 10, 15, 30 and 60 minutes, and free protein was quantified using a modified Bradford assay. Data are represented as mean values ± s.d. (n = 3).

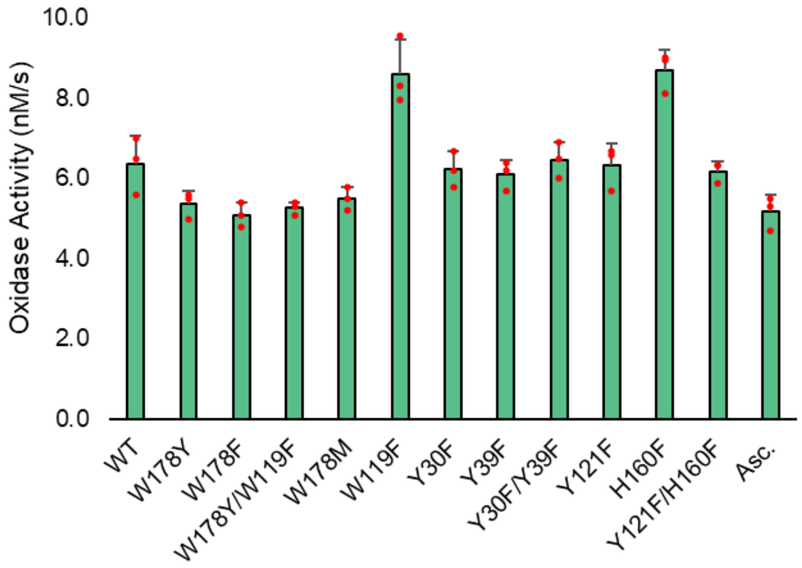

Supplementary Figure 10. Oxidase activity of *Sm*AA10A variants (2 μM), as determined with the Amplex Red assay performed in 50 mM Tris, pH 8.0, using 1 mM ascorbate as reductant. All initial rates (derived from linear progress curves for formation of resorufin) obtained are just above the ascorbate only control, indicating very slow H_2O_2 production for all variants tested. Note that H_2O_2 production by the LPMO is low compared to H_2O_2 production resulting from auto-oxidation of ascorbate and that he contribution of the LPMO will be even lower in reactions with an LPMO substrate, in which the LPMO oxidase activity will be surpressed[3, 7, 8]. The bars represent the mean value ± s.d. (n = 3; independent measurements are shown as red dots).

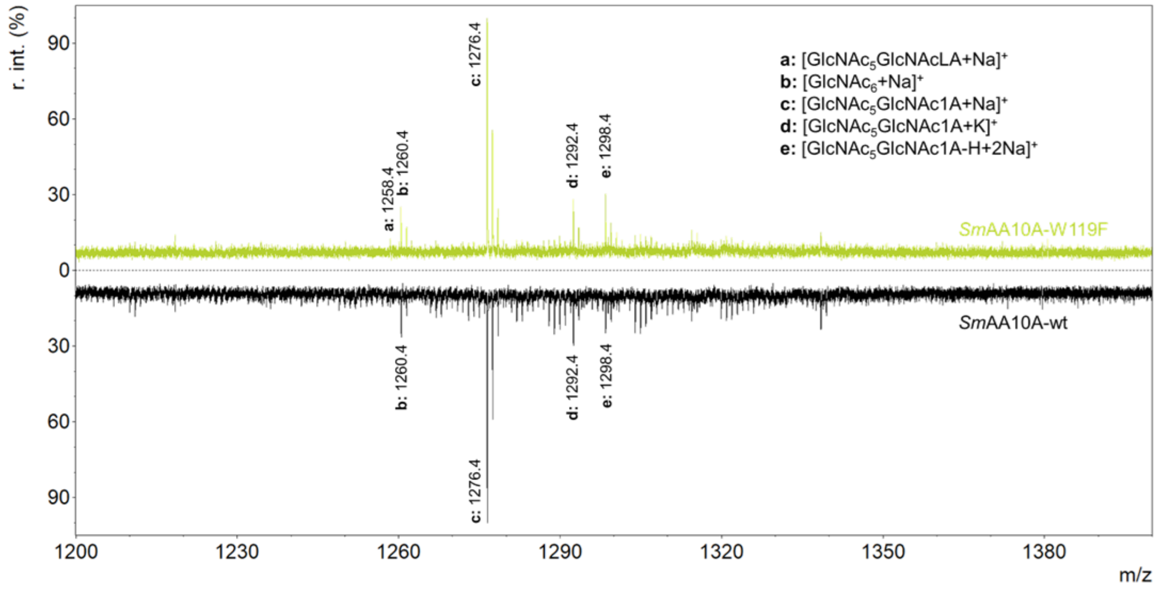

Supplementary Figure 11. MALDI-TOF analysis of chitin oxidation by wild-type *Sm*AA10A and the W119F variant. The figure shows signals corresponding to soluble native and oxidized LPMO products within the DP6 range (DP, degree of polymerization). The products were observed as sodium or potassium adducts or as sodium adducts of sodium salts, as indicated (GlcNAc, *N*-acetylglucosamine; GlcNAc1A, corresponding aldonic acid; GlcNAcLA, corresponding lactone). Substrate solubilization did not occur in control reactions containing LPMO but lacking the reductant. The reactions were run for 60 minutes in 50 mM Tris, pH 8.0, containing 10 g/L ß-chitin, 1 mM ascorbate, 300 µM H_2O_2 and 1 µM LPMO, at 37 °C. Note that both *Sm*AA10A and the W119F variant show similar substrate oxidation patterns (i.e., strict C1-oxidation of chitin). Signal normalization was based on the max signal observed for each enzyme (the signal for ion c, the sodium adduct of the aldonic acid). The figure was created using mMass 5.5.0 software.

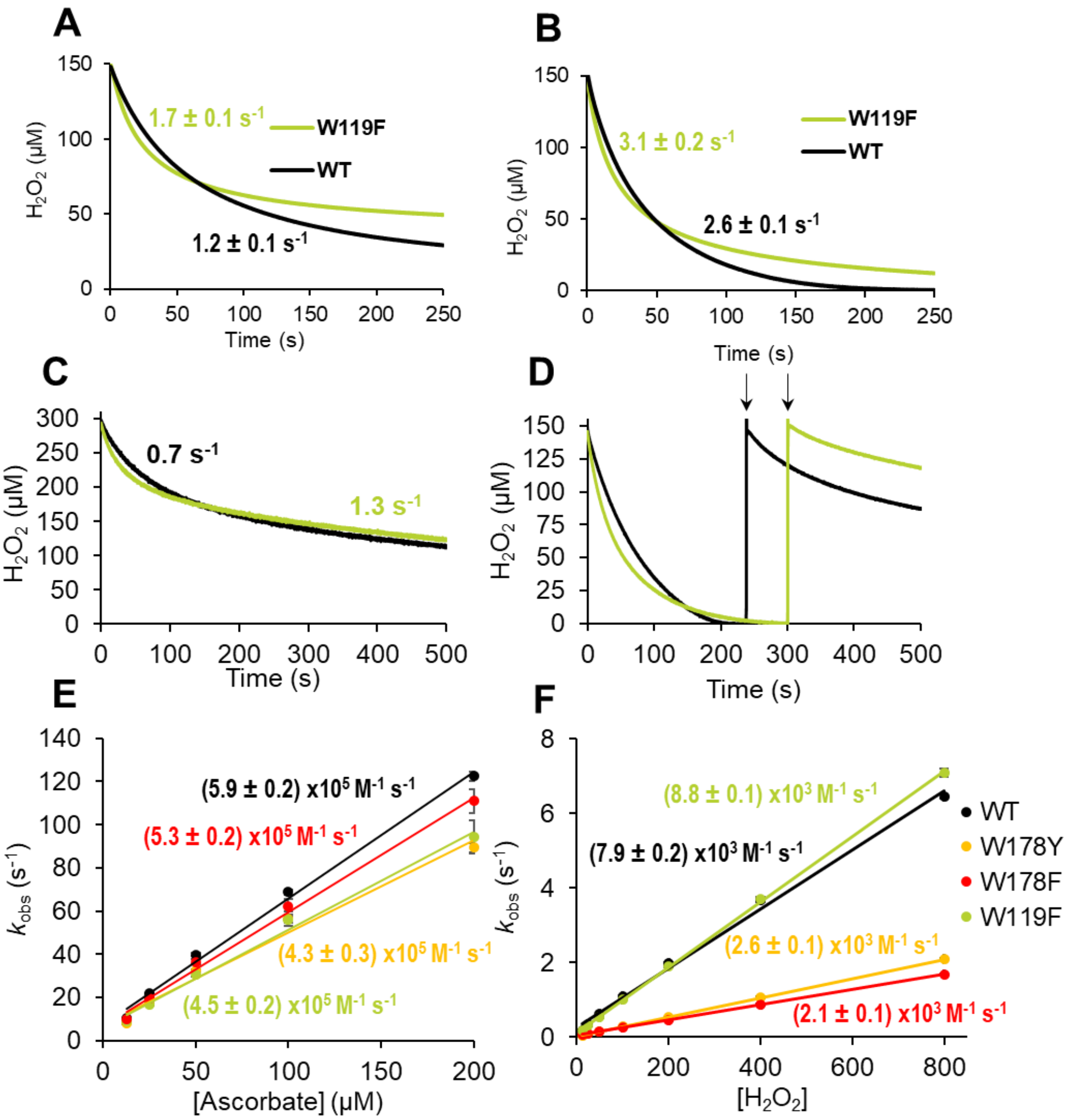

Supplementary Figure 12. Kinetic characterization of the W119F variant of *Sm*AA10A using an H_2O_2 sensor and stopped-flow fluorescence spectroscopy. A-C: Consumption of H_2O_2 during incubation of β-chitin (10g L^{-1}) with the LPMO (1 μM) in 50 mM Tris, pH 8.0, at 37 °C, using different reaction set-ups: **A**, 150 μM H_2O_2 and 50 μM ascorbate (this is the same Figure as Fig. 5A in the main manuscript); **B**, 150 μM H_2O_2 and 1 mM ascorbate; **C**, 300 μM H_2O_2 and 1 mM ascorbate, 30°C. The progress curves reflect three components: productive consumption of H_2O_2 (i.e. cleavage of chitin), minor abiotic depletion of H_2O_2 and off-pathway peroxidase-like consumption of H_2O_2 by the LPMO, which will be considerable at these high H_2O_2 levels and lead to enzyme inactivation. The time traces shown were corrected for background abiotic consumption of H_2O_2 and the background peroxidase reaction, assuming that this latter reaction is not suppressed by the presence of substrate, which seems reasonable since the reactions were done at high, damaging, H_2O_2 concentrations. The indicated turnover numbers (s^{-1}) were obtained by fitting the first 15 s of the H_2O_2 time traces with a linear function (R2 > 0.98 or higher). When not correcting for the peroxidase background these turnover numbers would be (value for W119F comes first): 2.74 and 2.02 s^{-1}, 4.48 and 3.49 s^{-1}, and 2.9 and 2.1 s^{-1}, for the experiments in panels **A**, **B** and **C**, respectively. Thus, in all

experiments, and no matter the background correction, the W119F variant consumed H_2O_2 faster than the WT. Note that these numbers imply that the background peroxidase activity of W119F was also consistently higher, compared to WT. Note also that the conditions in panel **C** are highly damaging, due to a very high H_2O_2 concentration, and that, while a reasonable linear fit was possible, the initial rates in fact are apparent initial rates with values that are severely affected by enzyme inactivation.

Inactivation of the LPMOs is evident from the fact that the baseline is not reached (i.e., no complete consumption of added H_2O_2). The curvatures in panels **A-C** show that, in all three cases, H_2O_2 consumption levels off faster for W119F, which shows that this variant is more readily damaged. Panel **D** incorporates an experiment identical to that shown in panel **B** (where the base line is reached) and shows what happens upon addition of another 150 μM of H_2O_2. The much lower rates observed in this second reaction cycle show that considerable enzyme inactivation had taken place in the first reaction cycle, as would be expected when using these levels of H_2O_2. Note that, in this second cycle, the initial rate of the W119F variant no longer surpasses that of the wild-type, underpinning that this variant suffered more damage in the first cycle. While the wildtype maintained approximately 33 ± 7 % of its initial activity, the W119F variant maintained less than 10 %.

The perhaps somewhat surprising dependency of the catalytic rates on the concentration of ascorbate is a consequence of the extreme conditions: when H_2O_2 levels are high, the peroxidase reaction will be prominent, which leads to consumption of ascorbate and, eventually, enzyme inactivation[9].

E-F: Second order rate constants for reduction with ascorbate (**E**) and reoxidation with H_2O_2 (**F**) of WT *Sm*AA10A and the variants W178Y, W178F and W119F in 50 mM Tris, pH 8.0, at 25 °C, determined by linear regression analysis of rates determined at different concentrations of ascorbate and H_2O_2, respectively. Data are presented as means ± s.d. (n = 3). Panel **E** shows that the rates of ascorbate-driven reduction are similar for WT, W119F, W178Y and W178F, while the reoxidation rate is 3 to 4-fold higher for those enzymes containing the W178 in close proximity to the copper (WT and W119F). This indicates that W178 is involved in the (substrate-independent) turnover of H_2O_2.

EPR Spectroscopy

Electron paramagnetic resonance (EPR) spectroscopy probes the geometric and electronic properties of paramagnetic centers, allowing for a selective and local inspection of the copper (II) active site in LPMOs. EPR is an extremely sensitive probe to any immediate structure changes of the copper active-site through the more distant mutations studied herein. The monocopper active site is coordinated by three nitrogen ligands, two *via* the imidazole nitrogen and terminal amino group of a bidentate *N*-terminal histidine, and the third nitrogen coordinates from the imidazole of a second histidine side chain (Histidine-brace). The T-shape coordination is highly conserved and allows for additional coordination of water or other solvent molecules (Supplementary Fig. 13b)[10].

The continuous-wave CW X-band (~9.46 GHz) EPR spectrum of WT in 50 mM Tris at pH 8.1 can be rationalized as a mixture of two components, **LPMO-1** and **LPMO-2**, Supplementary Fig. 13a. While component **LPMO-1** exhibits a broad EPR response with a rhombic **g**-tensor, **LPMO-2** has a distinctly axial spectrum with resolved ^{14}N superhyperfine along $g_{\perp}$. Simulation of the spectrum yields estimates on the relative ratio of both components of 55 % **LPMO-1** and 45 % **LPMO-2**. The full EPR simulation parameters are reported in the caption of Supplementary Figure 13.

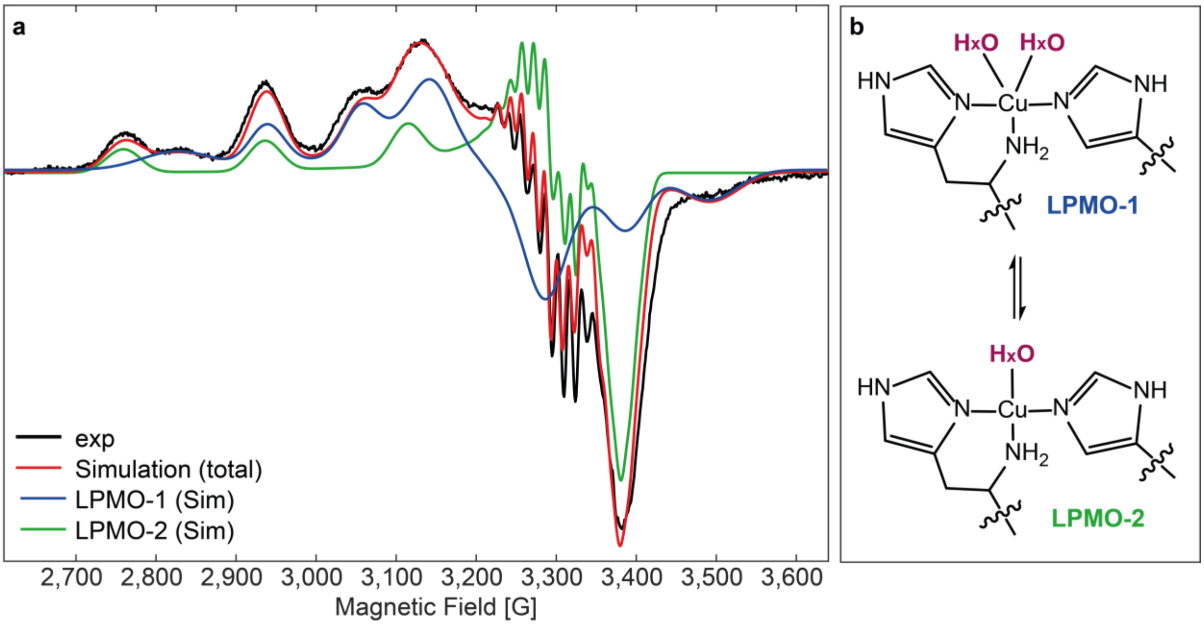

Supplementary Figure 13: EPR spectrum of wild type enzyme. a: CW X-band (9.46 GHz) EPR spectrum of *Sm*AA10A at pH 8.1 in 50 mM Tris obtained at 100 K (in black) with total simulation in red. The simulation of the individual components **LPMO-1** and **LPMO-2** are depicted in blue and green. EPR simulation parameters: For **LPMO-1**: **g** = [2.252 2.096 2.020]; **A**(^{63}Cu) = [360 40 280] MHz; **A**(^{14}N) = [30 30 40; 30 30 40; 30 40 30] MHz; line width = 2 mT; **AStrain** = [200 0 120] MHz. For **LPMO-2**: **g** = [2.234 2.050 2.045]; **A**(^{63}Cu) = [544 55 40] MHz; **A**(^{14}N) = [35 45 35; 35 45 35; 35 35 45] MHz; line width = 1.0 mT; **HStrain** = [70 0 40] MHz. Weighting of the individual components:

55 % (**LPMO-1**) : 45 % (**LPMO-2**). Spectrometer conditions are described in the Methods. **b**: histidine brace in LPMOs showing the pH-dependent equilibrium between component **LPMO-1** and **LPMO-2**.

This agrees with previous observations for multiple AA10 LPMOs that each exhibit an EPR spectrum consisting of two species, with both a rhombic and an axial EPR component[11–15]. It was shown for several LPMOs, by EPR spectroscopy and computational studies, that the different EPR responses (components) are attributed to differences in the number of aquo/hydroxo ligands. For rhombic EPR spectra, a five-coordinate copper is expected, with two coordinating water species, while the axial EPR spectra are assigned to a four coordinate species. These water coordination differences have been studied by varying the sample's pH or binding of substrate[14–16].

The ^{14}N superhyperfine pattern along $g_{\perp}$ in **LPMO-2** is well reproduced by the simulation of the EPR with the inclusion of three strongly coupled nitrogens, A ~ 35 – 45 MHz, confirming 3*N*-coordination environment of the histidine brace.

To investigate potential changes in the active site upon mutation, CW X-band EPR spectra of several variants (W119F, W178Y, W178M, W178F, W178Y/W119F) were measured, Supplementary Fig. 14. Overall, spectra with very similar responses were obtained for all variants. Most importantly, the ^{14}N superhyperfine pattern (~ 3,200 G – 3,360 G), including both the number of lines and the magnitude of the splitting, is consistent among the samples. As described earlier for the simulation of **LPMO-2**, the ^{14}N hyperfine pattern is only reproduced by the inclusion of three strongly coupled nitrogens (i.e. two coordinating imidazoles and the *N*-terminus amine). The persistent nitrogen hyperfine pattern compared to the wild type enzyme confirms both the integrity of the Histidine-Brace and identical local copper coordination environments for the various variants, Supplementary Figs. 14b and 14c. However, W178M exhibits a slightly larger line width, which precludes the observation of the ^{14}N superhyperfine splittings at high field positions. Nevertheless, g-values and the copper hyperfine remain constant, indicating no significant changes of the copper coordination sphere.

For the various variants studied, slight intensity shifts along $g_{\perp}$ can be observed, that are attributed to a change in the **LPMO-1**:**LPMO-2** ratio of the two components. These changes may be the results of minor differences in sample pH, unknown second sphere effects that may influence water binding through altered hydrogen bonding networks and/or introducing additional strains (disorder) resulting in broadened spectra. Nevertheless, the influence of these minor changes is expected to be neglectable for the activity of the respective variants, particularly as the EPR spectra of subcomponent **LPMO-2** offer a high-resolution glimpse of the histidine brace and its uniformity in the presence of the distant aromatic mutations.

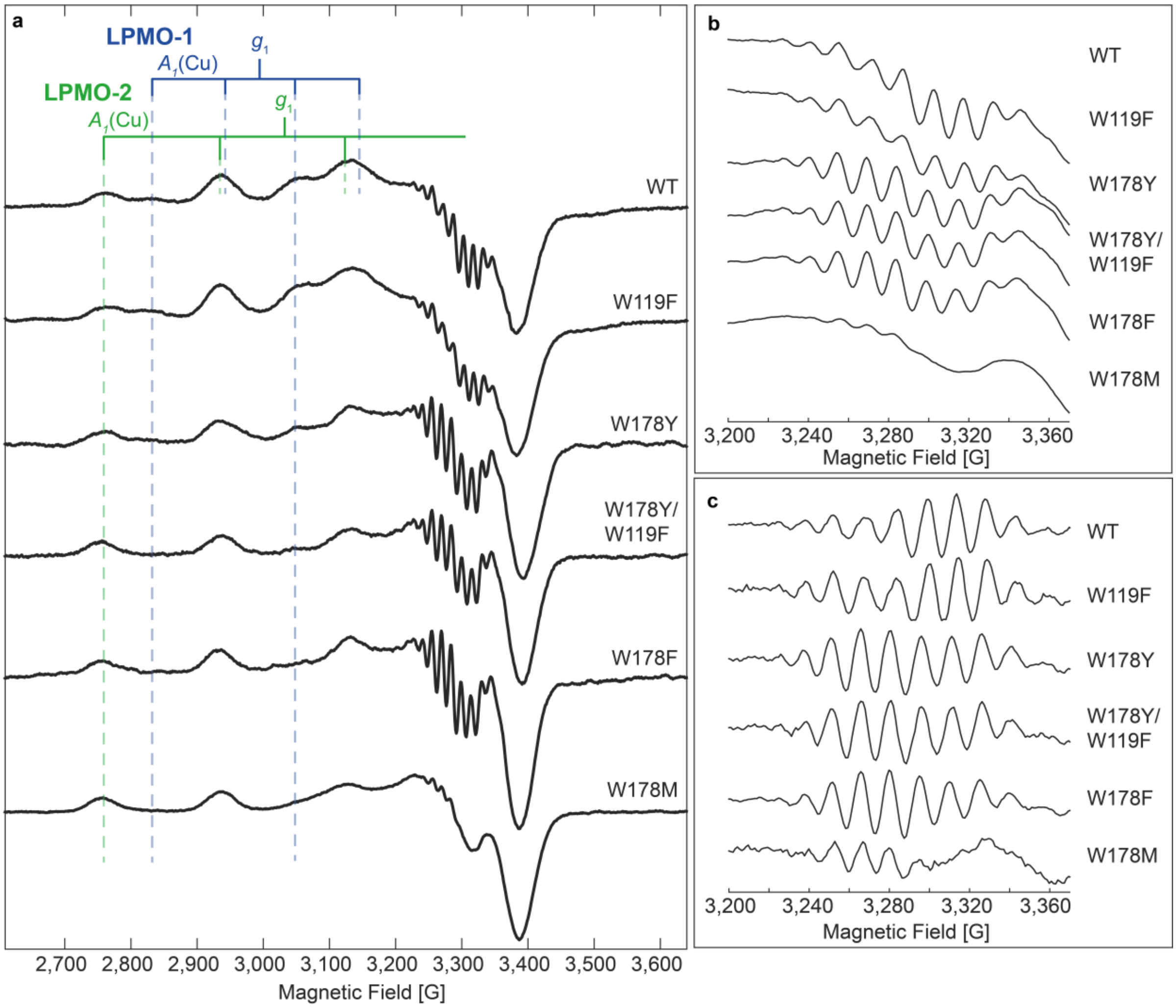

Supplementary Figure 14: EPR spectra of SmAA10A and its variants. CW X-band EPR spectra of *Sm*AA10A and its variants at pH 8.1 in 50 mM Tris obtained at 100 K (**a**) with the copper hyperfine along g_1 for both components **LPMO-1** (blue) and **LPMO-2** (green) marked by the colored lines. An enlargement of the resolved ^{14}N superhyperfine in the $g_\perp$ region of component **LPMO-2** (**b**) and the derivative spectra (**c**) are depicted to emphasize similarities between variants. Spectrometer conditions are described in the Methods.

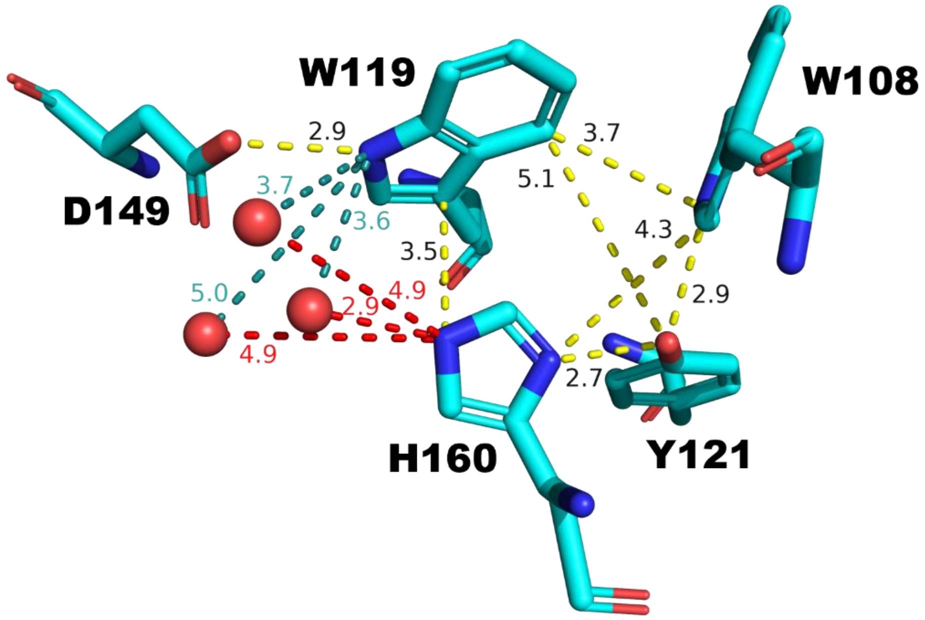

Supplementary Figure 15. The environment of H160 and W119 in SmAA10A WT. Distances were measured using PyMol and the crystal structure of *Sm*AA10A (pdb: 2bem, chain C). Only the side chains of selected amino acids, potentially involved in hole hopping and proton transfer, are shown; see the main text for details. The other two chains in 2bem also show crystallographic waters close to His160 and Trp119.

Supplementary Table 1. Strategy to generate each variant gene and list of primers used in this study. Experimental details can be found in the main manuscript's Methods section.

Variant	Cloning	Primers pair, sense and antisense (5' →3')
W178Y/W119F	Gene fragment ordered from Twist Biosciences.	
W178Y	QuickChange mutagenesis of W178Y/W119F, reverting W119F to native W119	CACCAGCTGGCGCTATTTCATCACCAAGCCGAAC<br>AAATAGCGACCGCTGGTGGTGCTGTGACGCGCGG
W178M	Gene fragment ordered from Twist Biosciences	
W178F	Quickchange mutagenesis of the WT gene.	GTGATCCTTGCCGTGTTCGACATAGCCGACACCG<br>CGGTGTCGGCTATGTCGAACACGGCAAGGATCAC
W119F	Quickchange mutagenesis of the WT gene.	CACCAGCTTTCGCTATTTCATCACCAAGCCGAAC<br>AAATAGCGAAAGCTGGTGGTGCTGTGACGCGCGG
Y30F	Quickchange mutagenesis of the WT gene.	CCACGGTTTTGTCGAATCGCCGGCCAGCCGCG<br>GATTCGACAAAACCGTGGGCATTCGCCTGTTG
Y39F	Quickchange mutagenesis of the WT gene.	CCGCGCCTTTCAGTGCAAACTGCAGCTCAACA<br>TTGCACTGAAAGGCGCGGCTGGCCGGCGATTC
Y30F/Y39F	Quickchange mutagenesis of Y30F with the same primers as for Y39F	
Y121F	Quickchange mutagenesis of the WT gene.	AGCTGGCGCTTTTTCATCACCAAGCCGAACTG<br>GATGAAAAAGCGCCAGCTGGTGGTGCTGTGAC
H160F	Quickchange mutagenesis of the WT gene.	GCCGCACAGGTCACCTTCCAGTGCAACATACC<br>GGTATGTTGCACTGGAAGGTGACCTGTGCGGC
Y121F/H160F	Quickchange mutagenesis of Y121F with the same primers as for H160F	
W108A	Quickchange mutagenesis of the WT gene.	TCCTTTACCGCGAAGCTGACCGCGCGTCACAGCACCACCAGCT<br>CAGCTTCGCGGTAAAGGAGTTCGGACCGGTTTTCAGGTTGAGC
W108M	Quickchange mutagenesis of the WT gene.	TCCTTTACCATGAAGCTGACCGCGCGTCACAGCACCACCAGCT<br>CAGCTTCATGGTAAAGGAGTTCGGACCGGTTTTCAGGTTGAGC
W108Y	Quickchange mutagenesis of the WT gene.	TCCTTTACCTACAAGCTGACCGCGCGTCACAGCACCACCAGCT<br>CAGCTTGTAGGTAAAGGAGTTCGGACCGGTTTTCAGGTTGAGC

Supplementary references

1 K. Huynh and C. L. Partch, "Analysis of protein stability and ligand interactions by thermal shift assay," *Curr. Protoc. Protein Sci.*, vol. 79, no. 1, pp. 28.9.1-28.9.14, Feb. 2015, doi: https://doi.org/10.1002/0471140864.ps2809s79.

2 O. Ernst and T. Zor, "Linearization of the bradford protein assay.," *J. Vis. Exp.*, no. 38, Apr. 2010, doi: 10.3791/1918.

3 R. Kittl, D. Kracher, D. Burgstaller, D. Haltrich, and R. Ludwig, "Production of four *Neurospora crassa* lytic polysaccharide monooxygenases in *Pichia pastoris* monitored by a fluorimetric assay," *Biotechnol. Biofuels*, vol. 5, no. 1, p. 79, 2012, doi: 10.1186/1754-6834-5-79.

4 A. A. Stepnov and V. G. H. Eijsink, "Looking at LPMO reactions through the lens of the HRP/Amplex Red assay.," *Methods Enzymol.*, vol. 679, pp. 163–189, 2023, doi: 10.1016/bs.mie.2022.08.049.

5 G. Vaaje-Kolstad *et al.*, "An oxidative enzyme boosting the enzymatic conversion of recalcitrant polysaccharides.," *Science*, vol. 330, no. 6001, pp. 219–222, Oct. 2010, doi: 10.1126/science.1192231.

6 L. Schwaiger, F. Csarman, H. Chang, O. Golten, V. G. H. Eijsink, and R. Ludwig, "Electrochemical monitoring of heterogeneous peroxygenase reactions unravels LPMO kinetics," *ACS Catal.*, pp. 1205–1219, Jan. 2024, doi: 10.1021/acscatal.3c05194.

7 A. A. Stepnov *et al.*, "Unraveling the roles of the reductant and free copper ions in LPMO kinetics," *Biotechnol. Biofuels*, vol. 14, no. 1, p. 28, 2021, doi: 10.1186/s13068-021-01879-0.

8 S. Brander *et al.*, "Scission of glucosidic bonds by a *Lentinus similis* lytic polysaccharide monooxygenases is strictly dependent on H_2O_2 while the oxidation of saccharide products depends on O_2," *ACS Catal.*, vol. 11, no. 22, pp. 13848–13859, Nov. 2021, doi: 10.1021/acscatal.1c04248.

9 S. Kuusk, V. G. H. Eijsink, and P. Väljamäe, "The 'life-span' of lytic polysaccharide monooxygenases (LPMOs) correlates to the number of turnovers in the reductant peroxidase reaction," *J. Biol. Chem.*, p. 105094, 2023, doi: https://doi.org/10.1016/j.jbc.2023.105094.

10 L. Ciano, G. J. Davies, W. B. Tolman, and P. H. Walton, "Bracing copper for the catalytic oxidation of C–H bonds," *Nat. Catal.*, vol. 1, no. 8, pp. 571–577, 2018, doi: 10.1038/s41929-018-0110-9.

11 A. K. Chaplin *et al.*, "Heterogeneity in the histidine-brace copper coordination sphere in auxiliary activity family 10 (aa10) lytic polysaccharide monooxygenases.," *J. Biol. Chem.*, vol. 291, no. 24, pp. 12838–12850, Jun. 2016, doi: 10.1074/jbc.M116.722447.

12 A. Munzone *et al.*, "Characterization of a bacterial copper-dependent lytic polysaccharide monooxygenase with an unusual second coordination sphere.," *FEBS J.*, vol. 287, no. 15, pp. 3298–3314, Aug. 2020, doi: 10.1111/febs.15203.

13 I. Serra *et al.*, "Activity and substrate specificity of lytic polysaccharide monooxygenases: An ATR FTIR-based sensitive assay tested on a novel species from *Pseudomonas putida*.," *Protein Sci.*, vol. 31, no. 3, pp. 591–601, Mar. 2022,

doi: 10.1002/pro.4255.

14 P. J. Lindley, A. Parkin, G. J. Davies, and P. H. Walton, “Mapping the protonation states of the histidine brace in an AA10 lytic polysaccharide monooxygenase using CW-EPR spectroscopy and DFT calculations,” *Faraday Discuss.*, vol. 234, no. 0, pp. 336–348, 2022, doi: 10.1039/D1FD00068C.

15 R. J. Gómez-Piñeiro *et al.*, “Decoding the ambiguous electron paramagnetic resonance signals in the lytic polysaccharide monooxygenase from *Photorhabdus luminescens*,” *Inorg. Chem.*, vol. 61, no. 20, pp. 8022–8035, May 2022, doi: 10.1021/acs.inorgchem.2c00766.

16 B. Bissaro, I. Isaksen, G. Vaaje-Kolstad, V. G. H. Eijsink, and Å. K. Røhr, “How a lytic polysaccharide monooxygenase binds crystalline chitin,” *Biochemistry*, vol. 57, no. 12, pp. 1893–1906, Mar. 2018, doi: 10.1021/acs.biochem.8b00138.
