# Supplementary material for: Mutational dissection of a hole hopping route in a lytic polysaccharide monooxygenase (LPMO)
Source: Nat Commun. 2024 May 10;15:3975. doi: 10.1038/s41467-024-48245-w (PMC11087555; doi:10.1038/s41467-024-48245-w)
Supplement: Supplementary file 3 — Reporting Summary [file 41467_2024_48245_MOESM3_ESM.pdf]

nature portfolio

Corresponding author(s): Ivan Ayuso-Fernandez & Vincent G.H. Eijsink

Last updated by author(s): Apr 12, 2024

Reporting Summary

Nature Portfolio wishes to improve the reproducibility of the work that we publish. This form provides structure for consistency and transparency in reporting. For further information on Nature Portfolio policies, see our Editorial Policies and the Editorial Policy Checklist.

Statistics

For all statistical analyses, confirm that the following items are present in the figure legend, table legend, main text, or Methods section.

n/a	Confirmed	
☐	☒	The exact sample size (*n*) for each experimental group/condition, given as a discrete number and unit of measurement
☒	☐	A statement on whether measurements were taken from distinct samples or whether the same sample was measured repeatedly
☒	☐	The statistical test(s) used AND whether they are one- or two-sided<br>*Only common tests should be described solely by name; describe more complex techniques in the Methods section.*
☒	☐	A description of all covariates tested
☒	☐	A description of any assumptions or corrections, such as tests of normality and adjustment for multiple comparisons
☐	☒	A full description of the statistical parameters including central tendency (e.g. means) or other basic estimates (e.g. regression coefficient) AND variation (e.g. standard deviation) or associated estimates of uncertainty (e.g. confidence intervals)
☒	☐	For null hypothesis testing, the test statistic (e.g. *F*, *t*, *r*) with confidence intervals, effect sizes, degrees of freedom and *P* value noted<br>*Give P values as exact values whenever suitable.*
☒	☐	For Bayesian analysis, information on the choice of priors and Markov chain Monte Carlo settings
☒	☐	For hierarchical and complex designs, identification of the appropriate level for tests and full reporting of outcomes
☒	☐	Estimates of effect sizes (e.g. Cohen's *d*, Pearson's *r*), indicating how they were calculated

Our web collection on statistics for biologists contains articles on many of the points above.

Software and code

Policy information about availability of computer code

Data collection	Chromeleon 7.0; Bio-Kine32 4.82; SkanIt RE 7.0.2; StepOne Software v2.3; flexControl version 3.4 (build 169.5), NOVA 1.1, https://github.com/IAyuso
Data analysis	Chromeleon 7.0; Bio-Kine32 4.82; Microsoft® Excel® for Microsoft 365 MSO (Version 2311 Build 16.0.17029.20140); Matlab R2023a; EasySpin v. 6.0.0-dev.51; flexControl version 3.4 (build 169.5); mMass 5.5.0; https://github.com/IAyuso

For manuscripts utilizing custom algorithms or software that are central to the research but not yet described in published literature, software must be made available to editors and reviewers. We strongly encourage code deposition in a community repository (e.g. GitHub). See the Nature Portfolio guidelines for submitting code & software for further information.

Data

Policy information about availability of data

All manuscripts must include a data availability statement. This statement should provide the following information, where applicable:

- Accession codes, unique identifiers, or web links for publicly available datasets
- A description of any restrictions on data availability
- For clinical datasets or third party data, please ensure that the statement adheres to our policy

All data generated in this study have been deposited in the figshare repository under accession https://doi.org/10.6084/m9.figshare.25261732. The crystal structure of SmAA10A (2bem) can be found at https://doi.org/10.2210/pdb2BEM/pdb. dbCAN database can be accessed and downloaded at https://bcb.unl.edu/dbCAN2/index.php.

Research involving human participants, their data, or biological material

Policy information about studies with human participants or human data. See also policy information about sex, gender (identity/presentation), and sexual orientation and race, ethnicity and racism.

Reporting on sex and gender	Not applicable.
Reporting on race, ethnicity, or other socially relevant groupings	Not applicable.
Population characteristics	Not applicable.
Recruitment	Not applicable.
Ethics oversight	Not applicable.

Note that full information on the approval of the study protocol must also be provided in the manuscript.

Field-specific reporting

Please select the one below that is the best fit for your research. If you are not sure, read the appropriate sections before making your selection.

☒ Life sciences ☐ Behavioural & social sciences ☐ Ecological, evolutionary & environmental sciences

For a reference copy of the document with all sections, see nature.com/documents/nr-reporting-summary-flat.pdf

Life sciences study design

All studies must disclose on these points even when the disclosure is negative.

Sample size	Sample sizes (number of replicate experiments) were chosen according to common practice in enzyme research and were such that standard deviations were small enough to allow determination of effects and trends. In most cases, a minimum of 3 independent replicates were carried out per experiment. Relevant examples in the LPMO field are: Bissaro B, Røhr ÅK, Müller G, Chylenski P, Skaugen M, Forsberg Z, Horn SJ, Vaaje-Kolstad G, Eijsink VGH. Oxidative cleavage of polysaccharides by monocopper enzymes depends on H2O2. Nat Chem Biol. 2017 Oct;13(10):1123-1128. doi: 10.1038/nchembio.2470. Epub 2017 Aug 28. PMID: 28846668/// Breslmayr E, Daly S, Požgajčić A, Chang H, Rezić T, Oostenbrink C, Ludwig R. Improved spectrophotometric assay for lytic polysaccharide monooxygenase. Biotechnol Biofuels. 2019 Dec 5;12:283. doi: 10.1186/s13068-019-1624-3. PMID: 31827611; PMCID: PMC6894463/// Chang, H., Gacias Amengual, N., Botz, A. et al. Investigating lytic polysaccharide monooxygenase-assisted wood cell wall degradation with microsensors. Nat Commun 13, 6258 (2022). https://doi.org/10.1038/s41467-022-33963-w
Data exclusions	No data was excluded from the analysis.
Replication	All experiments were performed in the number of replicates indicated in the manuscript (independent experiments, n = 3, all attempts successful). The data represented is the mean of the independent experiments, all data is included in means (no data excluded), and represented as means +/- standard deviation.
Randomization	Randomization is not relevant for this study since selection bias is not an issue.
Blinding	Not relevant since the bias that blinding prevents is not an issue in our study.

Reporting for specific materials, systems and methods

We require information from authors about some types of materials, experimental systems and methods used in many studies. Here, indicate whether each material, system or method listed is relevant to your study. If you are not sure if a list item applies to your research, read the appropriate section before selecting a response.

Materials & experimental systems

n/a	Involved in the study
☒	☐ Antibodies
☒	☐ Eukaryotic cell lines
☒	☐ Palaeontology and archaeology
☒	☐ Animals and other organisms
☒	☐ Clinical data
☒	☐ Dual use research of concern
☒	☐ Plants

Methods

n/a	Involved in the study
☒	☐ ChIP-seq
☒	☐ Flow cytometry
☒	☐ MRI-based neuroimaging

Plants

Seed stocks	*Report on the source of all seed stocks or other plant material used. If applicable, state the seed stock centre and catalogue number. If plant specimens were collected from the field, describe the collection location, date and sampling procedures.*
Novel plant genotypes	*Describe the methods by which all novel plant genotypes were produced. This includes those generated by transgenic approaches, gene editing, chemical/radiation-based mutagenesis and hybridization. For transgenic lines, describe the transformation method, the number of independent lines analyzed and the generation upon which experiments were performed. For gene-edited lines, describe the editor used, the endogenous sequence targeted for editing, the targeting guide RNA sequence (if applicable) and how the editor was applied.*
Authentication	*Describe any authentication procedures for each seed stock used or novel genotype generated. Describe any experiments used to assess the effect of a mutation and, where applicable, how potential secondary effects (e.g. second site T-DNA insertions, mosiacism, off-target gene editing) were examined.*
